# Neurosurgical treatment of pediatric brain tumors - results from a single center multidisciplinary setup

**DOI:** 10.1007/s00381-023-06123-8

**Published:** 2023-09-21

**Authors:** A. Schaumann, C. Hammar, S. Alsleben, M. Schulz, A. Grün, E. Lankes, A. Tietze, Arend Koch, P. Hernáiz Driever, U.-W. Thomale

**Affiliations:** 1 Charité–Universitätsmedizin Berlin, corporate member of Freie Universität Berlin and Humboldt-Universität zu Berlin, Pediatric Neurosurgery, Augustenburger Platz 1, 13353 Berlin, Germany; 2 Charité–Universitätsmedizin Berlin, corporate member of Freie Universität Berlin and Humboldt-Universität zu Berlin, Department for Radiation Oncology and Radiotherapy, Augustenburger Platz 1, 13353 Berlin, Germany; 3 Charité–Universitätsmedizin Berlin, corporate member of Freie Universität Berlin and Humboldt-Universität zu Berlin, Department of Pediatric Endocrinology and Diabetes, Augustenburger Platz 1, 13353 Berlin, Germany; 4 Charité–Universitätsmedizin Berlin, corporate member of Freie Universität Berlin and Humboldt-Universität zu Berlin, Institute of Neuroradiology, Augustenburger Platz 1, 13353 Berlin, Germany; 5 Charité - Universitätsmedizin Berlin, corporate member of Freie Universität Berlin and Humboldt-Universität zu Berlin, Institute of Neuropathology, Berlin, Germany; 6 Charité–Universitätsmedizin Berlin, corporate member of Freie Universität Berlin and Humboldt-Universität zu Berlin, Department of Pediatric Hematology and Oncology, Augustenburger Platz 1, 13353 Berlin, Germany

**Keywords:** Pediatric brain tumor, Neurologic deficits, Medulloblastoma, Pilocytic Astrocytoma, Ependymoma, High grade glioma, Extent of resection, Transfusion rates, Morbidity

## Abstract

**Objective:**

The challenge of pediatric brain tumor surgery is given due to a relative low prevalence but high heterogeneity in age, localization, and pathology. Improvements of long-term overall survival rates were achieved during the past decades stressing the importance of a multidisciplinary decision process guided by a national treatment protocol. We reviewed the entire spectrum of pediatric brain tumor surgeries from the perspective of an interdisciplinary pediatric neuro-oncology center in Germany.

**Methods:**

Every patient who underwent brain tumor surgery from January 2010 to June 2017 in our Pediatric Neurosurgery department was retrospectively included and evaluated regarding the course of treatment. Perioperative data such as tumor localization, timing of surgery, extent of resection, neuropathological diagnosis, transfusion rates, oncologic and radiation therapy, and neurological follow-up including morbidity and mortality were evaluated.

**Results:**

Two hundred ninety-three pediatric brain tumor patients were applicable (age: 8.28 ± 5.62 years, 1.22:1.0 m:f). A total of 531 tumor surgical interventions was performed within these patients (457 tumor resections, 74 tumor biopsies; mean interventions per patient 1.8 ± 1.2). Due to a critical neurologic status, 32 operations (6%) were performed on the day of admission. In 65.2% of all cases, tumor were approached supratentorially. Most frequent diagnoses of the cases were glial tumors (47.8%) and embryonal tumors (17.6%). Preoperative planned extent of resection was achieved in 92.7%. Pre- and postoperative neurologic deficits resolved completely in 30.7%, whereas symptom regressed in 28.6% of surgical interventions. New postoperative neurologic deficit was observed in 10.7%, which resolved or improved in 80% of these cases during 30 days. The mortality rate was 1%.

**Conclusion:**

We outlined the center perspective of a specialized pediatric neuro-oncological center describing the heterogeneous distribution of cases regarding age-related prevalence, tumor localization, and biology, which requires a high multidisciplinary expertise. The study contributes to define challenges in treating pediatric brain tumors and to develop quality indicators for pediatric neuro-oncological surgery. We assume that an adequate volume load of patients within a interdisciplinary infrastructure is warranted to aim for effective treatment and decent quality of life for the majority of long-term surviving pediatric tumor patients.

**Supplementary Information:**

The online version contains supplementary material available at 10.1007/s00381-023-06123-8.

## Introduction

Brain tumors are the second most frequent malignancies during childhood [1]. Approximately 2–4 patients/100,000 inhabitants are newly diagnosed per year in Germany, i.e., approximately 500 children and adolescents [2–4]. Management of pediatric brain tumor patients is a multidisciplinary challenge in which brain surgery is often the primary intervention for tumor diagnosis and treatment. Surgical interventions should be carried out in accordance with current recommendations and guidelines and require a well-coordinated multidisciplinary team due to the complexity of specified treatment protocols. National networks such as the German brain tumor network (HIT Netzwerk der Gesellschaft für Pädiatrische Onkologie und Hämatologie, GPOH) develop and consent on strategies for treatment of pediatric brain tumors by interdisciplinary study committees.

Over the last two decades, only few pediatric brain tumor case series were published, reporting about center-specific treatment concepts [5–7]. This encourages a more recent data analysis on incidence and surgical treatment modalities of pediatric brain tumors in the context of improved surgical techniques due to technical advances. Postoperative morbidity can be an important obstacle for an effective treatment as it may influence long-term quality of life in brain tumor survivors, especially as the 10-year overall survival rate is approximately 80% [1, 8]. Weighing risks and benefits of surgical and non-surgical treatment options among each other is mandatory; therefore, establishing surgical protocols is crucial. The appropriate surgical strategy in this challenging patient cohort is based on the multidisciplinary team (MDT) decision to either achieve gross total resection, tumor debulking, or only tumor biopsy, as operative risks are to be minimized for each individual patient. Appropriate communication with patients and caregivers are nevertheless equally essential to manage the expectations regarding risks and benefits. Therefore, the perspective of the treating surgical center is important in order to investigate and improve treatment standards as well as quality indicators.

The aim of this retrospective single-center study is to display data regarding the pediatric neurosurgical treatment paradigm of children and adolescence with brain tumors focusing on the presented case range and the surgical and neurological outcome. Thereby, we aim to outline the possible challenges and healthcare needs as well as to define quality indicators for establishing specialized pediatric brain tumor care.

## Methods

We retrospectively identified all patients who underwent at least one brain tumor surgery in our Pediatric Neurosurgery department between January 2010 and June 2017. The entire course of the brain tumor surgeries was traced back in these patients from the year 2000 on. Data were retrieved from the medical charts and filed in a specifically designed brain tumor surgery database (Filemaker Pro 19; Claris International, CA, USA). The database entry was completed until 02/2020. In addition to basic clinical and neurosurgical data, non-surgical oncologic treatment modalities and clinical follow-up data were collected over the entire course of treatment.

Follow-up time was calculated from the date of first surgery to the date of last contact with our institution. Patients were allocated into five groups depending on age at each surgery (0–1 year of age: infants; 1–3 years of age: toddler; 3–6 years of age: kindergarten; 6–12 years of age: prepubescent; 12–18 years of age: pubescent; > 18 years of age: young adults). Distance of patients’ residence to our pediatric neurosurgery department was recorded and divided into three distinctive groups (< 50 km – “regional,” 50–500 km – “over-regional,” > 500 km – “distant”).

### Clinical management and course of patients

Patients presenting to a hospital with symptoms potentially indicating an intracranial mass lesion were examined by a pediatric neurologist or pediatric neurosurgeon. Following clinical work up and a cranial/spinal MRI scan to confirm the diagnosis, patients’ family were informed by the pediatric neurosurgeon and/or pediatric neuro-oncologist, while oncological psychology support was offered in parallel to the families. The case was discussed in the multidisciplinary oncological team (MDT) board (consisting of Pediatric Neuro-oncology, Pediatric Neuroradiology, Pediatric Neurosurgery, Pediatric Endocrinology and radiation therapists). In urgent circumstances, the interdisciplinary decisions were accomplished by a telephone conference. The board’s recommendations were presented to patients and their caregivers, and the proposed surgical treatment was defined outlining the benefits as well as possible risks of intervention. After obtaining consent to the planned surgery, interventions were performed by one of the senior pediatric neurosurgeon in charge. Urgency of the operations was determined as follows: “N0,” representing a medical urgency with immediate surgical intervention necessity; “N1,” surgery needed in the next available surgical room; “N2,” surgery within 6 h; “N3,” surgery within 12 h; “N4,” surgery within 24 h; and “N5,” elective surgery. All surgeries were accomplished under general anesthesia led by a specialized pediatric anesthesiology team. Routine antibiotic treatment was given before the surgery, using a second generation cephalosporine (cefuroxime, 50 mg/kg bodyweight) or alternatively clindamycin (10 mg/kg bodyweight) in case of a pre-existing contraindication. Corticosteroids were not given routinely but were indicated at the discretion of the pediatric neurosurgeon in charge in lesions with extended edema or striking mass effect. Postoperatively, patients were admitted to the intensive care unit, the intermediate care unit, or the post anesthesia care unit for at least 24 h. Postoperative MRI scans were retrieved within the first 48 h postoperatively to determine the extent of resection and exclude postsurgical abnormalities. Extent of tumor resection was determined based on an international consented classification [9] from pediatric neurosurgeon perspective (S1: macroscopically complete resection; S2 remnant of tumor rim, S3: remnant of relevant tumor mass and S4: biopsy only) as well as from the neuro-radiologist perspective according to MR imaging (R1: complete resection, R2: rim of tumor remnant < 5 mm; R3: relevant tumor remnant > 5 mm in diameter and R4: no change in tumor volume—biopsy only). These resection parameters were combined to the extent of resection classification types as follows: type I (total; S1&R1), type IIa (subtotal; S2&R1), type IIb (subtotal; S1&R2), type IIc (subtotal; S2&R2), type III (partial; S1/2/3&R3) and type IV (biopsy only, S4&R4)) [10]. The preoperatively anticipated extent of resection (as defined in the MDT) was compared to the postoperatively achieved extent of resection.

Intraoperatively tumor samples were taken and sent as “fresh tissue” [11] directly to our institute for neuropathology for primary diagnosis according to the WHO classification of 2007 [12] and 2016 [13], respectively. The recent 2021 edition [14–16] was not yet available during the study period of this patient cohort. Reference neuropathology examinations were obtained on routine basis in the specified neuropathology reference center (specific diagnosis related neuropathology reference center within the German pediatric neuro-oncological network).

For practical reasons, the histopathologic diagnosis was grouped into the following cluster: group 1a “LGG,” group 1b “HGG,” group 2 “ependymoma,” group 3 “embryonal tumors,” group 4 “craniopharyngioma,” and group 5 “other” (Table 1, for further detailed information: supplementary tables 1 and 3).

**Table 1:** Patient characteristics as divided in age distribution

**study overview**	**0–1 year**		**> 1–3 years**		**> 3–6 years**		**> 6–12 years**		**> 12–18 years**		**> 18 years**		**TOTAL**	
**item**	**n**	**%**	**n**	**%**	**n**	**%**	**n**	**%**	**n**	**%**	**n**	**%**	**n**	**%**
**number of patients**	16	5.5%	37	12.6%	64	21.8%	95	32.4%	64	21.8%	17	5.8%	293	100%
**Sex (m/f)**	5/11	31%/69%	21/16	57%/43%	35/29	55%/45%	56/39	59%/41%	35/29	55%/45%	9/8	53%/47%	161/132	55%/45%
**OP-cases**	32	6.0%	83	15.6%	111	20.9%	157	29.6%	122	23.0%	26	4.9%	531	100%
**primary operation**	18	56.3%	50	60.2%	54	48.6%	77	49.0%	46	37.7%	4	15.4%	249	46.9%
**internal revision**	12	37.5%	28	33.7%	51	45.9%	64	40.8%	65	53.3%	20	76.9%	240	45.2%
**external revision**	2	6.3%	5	6.0%	6	5.4%	16	10.2%	11	9.0%	2	7.7%	42	7.9%
**mean age at surgery (a)**	0.44 (±0.25)		2.08 (±0.61)		4.46 (±0.89)		8.74 (±1.71)		14.98 (±1.80)		19.78 (±1.54)		8.29 (±5.62)	
**tumor localization**														
**hemispheric**	13	40.6%	32	38.6%	24	21.6%	40	25.5%	39	32.0%	10	38.5%	158	29.8%
**frontal**	2	6.3%	17	20.5%	7	6.3%	11	7.0%	8	6.6%	5	19.2%	50	9.4%
**temporal**	6	18.8%	3	3.6%	9	8.1%	23	14.6%	17	13.9%	3	11.5%	61	11.5%
**parietal**	5	15.6%	9	10.8%	8	7.2%	3	1.9%	9	7.4%	2	7.7%	36	6.8%
**occipital**	0	0.0%	3	3.6%	0	0.0%	3	1.9%	5	4.1%	0	0.0%	11	2.1%
**sella region**	1	3.1%	14	16.9%	20	18.0%	29	18.5%	15	12.3%	5	19.2%	84	15.8%
**suprasellar**	1	3.1%	13	15.7%	18	16.2%	28	17.8%	10	8.2%	2	7.7%	72	13.6%
**sellar**	0	0.0%	1	1.2%	2	1.8%	1	0.6%	5	4.1%	3	11.5%	12	2.3%
**basal ganglia**	0	0.0%	4	4.8%	5	4.5%	12	7.6%	15	12.3%	4	15.4%	40	7.5%
**basal ganglia**	0	0.0%	0	0.0%	0	0.0%	1	0.6%	11	9.0%	3	11.5%	15	2.8%
**Thalamus**	0	0.0%	4	4.8%	5	4.5%	11	7.0%	4	3.3%	1	3.8%	25	4.7%
**ventricular system**	10	31.3%	17	20.5%	20	18.0%	26	16.6%	24	19.7%	6	23.1%	103	19.4%
**lat. ventricles**	3	9.4%	8	9.6%	6	5.4%	6	3.8%	7	5.7%	0	0.0%	30	5.6%
**third ventricle**	6	18.8%	2	2.4%	1	0.9%	1	0.6%	8	6.6%	1	3.8%	19	3.6%
**fourth ventricle**	1	3.1%	7	8.4%	13	11.7%	19	12.1%	9	7.4%	5	19.2%	54	10.2%
**infratentorial**	6	18.8%	16	19.3%	36	32.4%	40	25.5%	27	22.1%	0	0.0%	125	23.5%
**cerebellar**	6	18.8%	12	14.5%	28	25.2%	33	21.0%	21	17.2%	0	0.0%	100	18.8%
**brain stem**	0	0.0%	4	4.8%	8	7.2%	7	4.5%	6	4.9%	0	0.0%	25	4.7%
**pineal**	2	6.3%	0	0.0%	2	1.8%	6	3.8%	1	0.8%	0	0.0%	11	2.1%
**miscellaneous**	0	0.0%	0	0.0%	4	3.6%	4	2.5%	1	0.8%	1	3.8%	10	1.9%
**TOTAL:**	32	100%	83	100%	111	100%	157	100%	122	100%	26	100%	531	100%
**Sx-approach**														
**supratentorial**	25	78.1%	60	72.3%	60	54.0%	96	61.1%	85	69.7%	20	76.9%	346	65.2%
**infratentorial**	7	21.9%	23	27.7%	51	46.0%	61	38.9%	37	30.3%	6	23.1%	185	34.8%
**extent of resection**														
**type I (total; S1/R1)**	9	28.1%	21	25.3%	37	33.3%	53	33.8%	35	28.7%	3	11.5%	158	29.8%
**type IIa (subtotal; S2/R2)**	0	0.0%	1	1.2%	2	1.8%	2	1.3%	1	0.8%	0	0.0%	6	1.1%
**type IIb (subtotal; S1/R2)**	5	15.6%	10	12.0%	14	12.6%	16	10.2%	12	9.8%	3	11.5%	60	11.3%
**type IIc (subtotal; S2/R2)**	2	6.3%	18	21.7%	12	10.8%	18	11.5%	10	8.2%	7	26.9%	67	12.6%
**type III (partial; S123/R3)**	7	21.9%	19	22.9%	33	29.7%	46	29.3%	51	41.8%	10	38.5%	166	31.3%
**biopsy (S4/R4)**	9	28.1%	14	16.9%	13	11.7%	22	14.0%	13	10.7%	3	11.5%	74	13.9%
**peri-OP blood transfusion**														
**necessary**	14	43.8%	29	34.9%	32	28.8%	15	9.6%	4	3.3%	0	0.0%	94	17.7%
**not necessary**	18	56.3%	54	65.1%	79	71.2%	142	90.4%	118	96.7%	26	100.0%	437	82.3%
**neuropathology**														
**group 1a (LGG)**	4	12.5%	20	24.1%	50	45.0%	70	44.6%	53	43.4%	8	30.8%	205	38.6%
**group 1b (HGG)**	6	18.8%	9	10.8%	6	5.4%	13	8.3%	12	9.8%	6	23.1%	52	9.8%
**group 2 (ependymoma)**	0	0.0%	6	7.2%	17	15.3%	10	6.4%	10	8.2%	0	0.0%	43	8.1%
**group 3 (embryonal tumors)**	10	31.3%	25	30.1%	20	18.0%	30	19.1%	10	8.2%	2	7.7%	97	18.3%
**group 4 (craniopharyngioma)**	0	0.0%	6	7.2%	3	2.7%	14	8.9%	14	11.5%	5	19.2%	42	7.9%
**group 5 (miscellaneous)**	12	37.5%	17	20.5%	15	13.5%	20	12.7%	23	18.9%	5	19.2%	92	17.3%
**adjuvant therapy**														
**Sx only**	18	56.3%	28	33.7%	28	25.2%	55	35.0%	64	52.5%	6	23.1%	199	59.9%
**mTx**	14	43.8%	45	54.2%	54	48.6%	54	34.4%	28	23.0%	11	42.3%	206	62.0%
**rTx**	0	0.0%	5	6.0%	19	17.1%	22	14.0%	18	14.8%	4	15.4%	68	20.5%
**rmTx**	0	0.0%	5	6.0%	10	9.0%	26	16.6%	12	9.8%	5	19.2%	58	17.5%
**follow up data**	(according to age at first surgery in domo)													
**CR**	6	37.5%	11	29.7%	27	42.1%	32	33.6%	22	34.4%	2	11.8%	100	34.1%
**SD**	7	43.8%	9	24.3%	21	32.8%	46	48.4%	28	43.8%	12	70.6%	123	42%
**PD**	0	0%	4	10.8%	8	12.5%	11	11.6%	10	15.6%	3	17.6%	36	12.3%
**death**	3	18.7%	13	35.1%	8	12.5%	6	6.3%	4	6.2%	0	0.0%	34	11.6%
**mean follow up time**	4.84a (±3.15)		3.82a (±3.93)		4.19a (±3.30)		4.49a (±3.40)		1.13a (±0.87)		4.35a (±2.41)		4.27a (±3.31)	

Pre- and postoperative neurologic deficits including possible additional complications were retrieved from the patients’ hospital charts and were categorized into subgroups according to type and duration. Further non-surgical treatment was given according to the MDT board recommendation, i.e., systemic medical treatment (mTx; e.g., chemotherapeutic, immunomodulatory agents such as monoclonal antibodies and targeted therapy), radiation therapy (rTx, e.g., photon or proton beam)), or a combination of both (rmTx). Alternatively, an observational strategy with follow-up MRI examinations (Sx only) was chosen.

Clinical examinations for follow-up were executed generally by the pediatric neuro-oncologist according to the recommended adjuvant therapy regimen and, if necessary, in combination with the pediatric neurosurgeon. Follow-up MRIs were performed and analyzed according to recent HIT and/or SIOPe protocols/guidelines [17], respectively. Some of the patients needed to undergo multiple operations due to several reasons such as unsatisfactory tumor resection, progression of remnant tumor, tumor recurrence, or indecisive neuropathologic results. Surgery associated mortality rate was calculated for the period of 30 days post-surgery.

### Statistical analysis

The absolute and relative frequencies for categorical variables, mean, and standard deviation for continuous variables are reported descriptively. Characteristics of individuals in total and by age group and surgery characteristics are presented with the units of observation, respectively. Differences in parts of the whole were analyzed using the chi-square test with the Graphpad Prism 9 (Graphstats Technologies, CA, USA).

## Results

### Patients characteristics

**Fig. 1:**
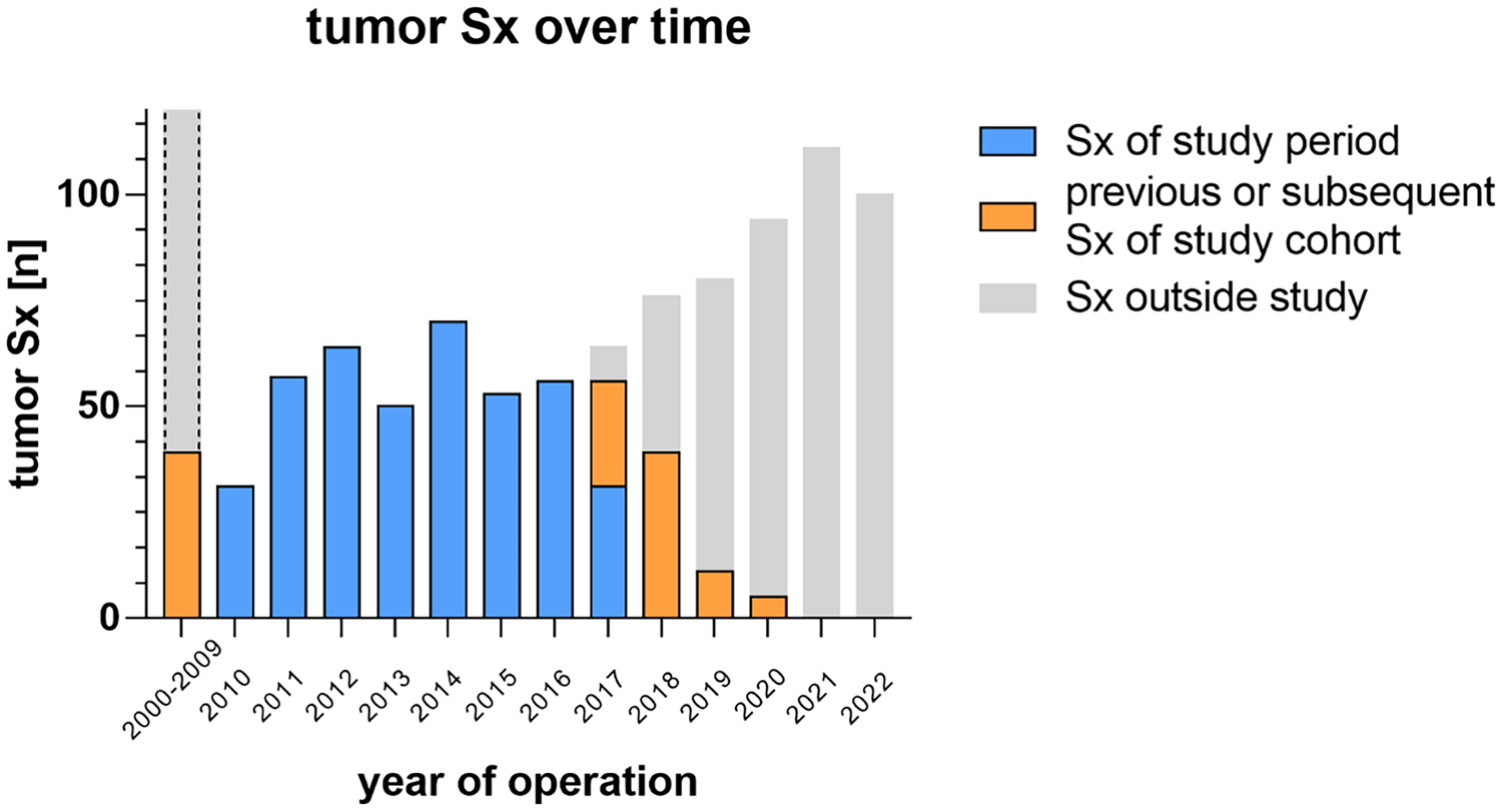
The number of treated tumor patients on a yearly basis during the study period between 01/2010 and 6/2017 as marked in blue. Subsequent surgeries of these patients were also evaluated from the time period between 2000 and 2020 as marked in orange. The number of tumor surgeries which are not included in the study are marked in gray

Two hundred ninety-three patients who were surgically treated in our department between 01/2010 and 06/2017 (7.5 years) were applicable for our study (161 males (54.9%), 132 females (45.1%)). The complete course of each patients’ surgical treatment between 01/2000 and 02/2020 (20 years) was monitored in a total of 531 surgical tumor-related interventions (457 tumor resections and 74 tumor biopsies; Fig. 1). The mean age was 8.28 ± 5.62 years, median 7.19 years, IQR 8.99 years (range: 2 days to 23.5 years at last surgical intervention). A slight male predominance was observed in every age group except for infants (Table 1).

The mean number of tumor surgeries per patient at our department was 1.8 ± 1.2 (range: 1–10, median 2, IQR 2). One hundred sixty-two patients received one operation (55.2%). Mean time between surgeries was 1.36 ± 1.74 years (range 0.01–11.2). Two hundred forty-nine operations (84.9%) were performed as primary intervention (first brain tumor surgery in patients` history) in our hospital of which 53.4% received only one surgery. Forty-four patients had previous surgeries in other institutions of which 65.9% received a single additional surgery at our institution (Table 1).

In terms of service coverage, the distance between the patient’s residence and hospital at the time of first surgery in our department was less than 50 km in 215 patients (73.4%), 50–500 km in 39 patients (13.3%), and more than 500 km in 39 patients (13.3%).

### Surgeries

Most of the surgeries were elective surgical interventions (405 surgeries, 76.3%). Thirty-two operations (6.0%) were performed as emergency procedures due to compromised clinical status of patients within 12 h after admission [18]. The surgical interventions were distributed as follows: N0: 1 case (0.2%), N1: 6 cases (1.2%), N2: 14 cases (2.8%), N3: 11 cases (2.2%), N4: 94 cases (18.7%), N5: 405 cases (76.3%).

**Fig. 2:**
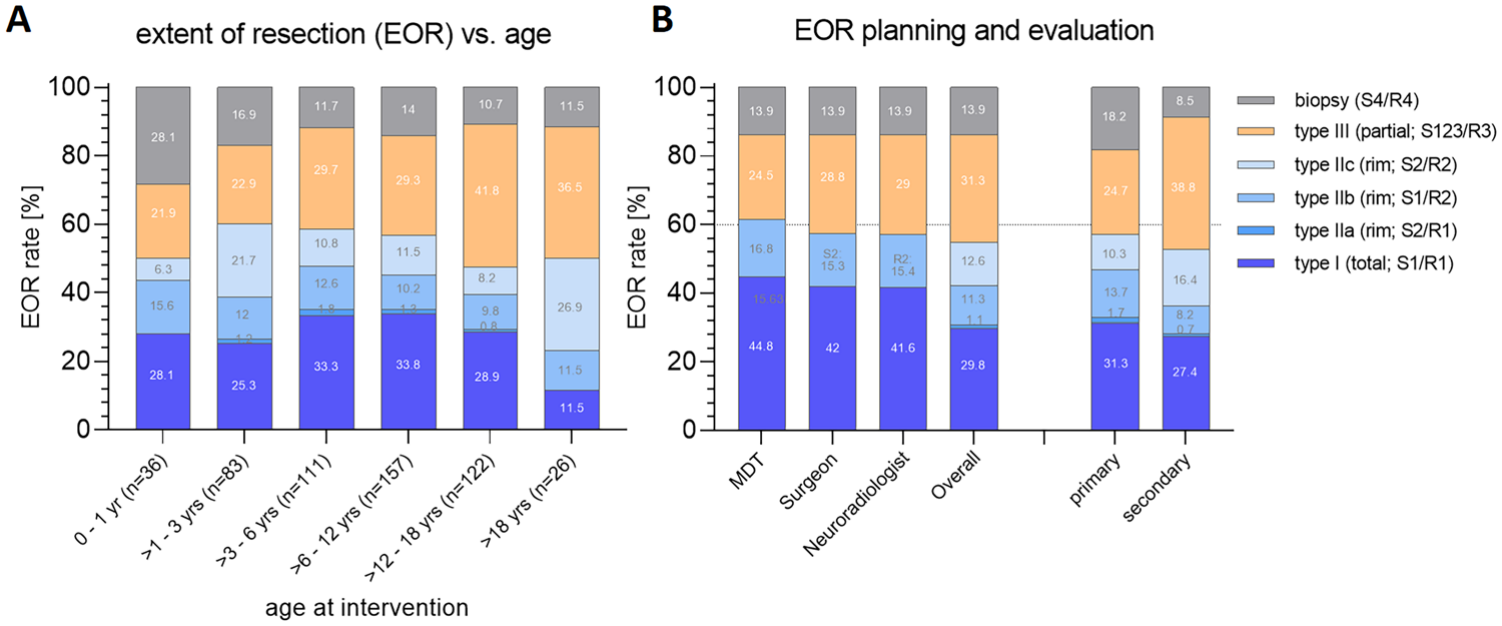
Age dependent variation in extent of resection (**A**) and comparison between MDT plan, surgeon evaluation, neuro-radiologist evaluation without significant differences in observed rates (**B**). A significant difference between EOR rates was observed between primary tumor surgeries compared to secondary tumor surgeries

The extent of tumor resection of all surgeries was as follows: type I in 158 cases (29.8%), type IIa in 6 cases (1.1%), type IIb in 60 cases (11.3%), type IIc in 67 cases (12.6%), type III in 166 cases (31.2%). In 74 cases (13.9%), a biopsy was performed. Resection grades did not relevantly differ between the age groups. In 92.7% of the cases, extent of resection was achieved according to MDT plan. In 39 surgeries, a lower EOR was achieved, and in 6 surgeries, a higher EOR was reached. No statistical differences between the rates of MDT plan compared to the surgeon’s or the neuro-radiologist’s evaluation was observed (Fig. 2).

Among patients who received a secondary tumor surgery, the EOR (type I: 27.4%, type II: 25.3%, type III in 38.8%, and type IV (biopsy): 8.5%) differed from patients who received primary tumor surgery (type I: 31.3%, type II: 25.4%, type III: 24.7%, and type IV (biopsy): 18.2%, *p* < 0.01).

**Fig. 3:**
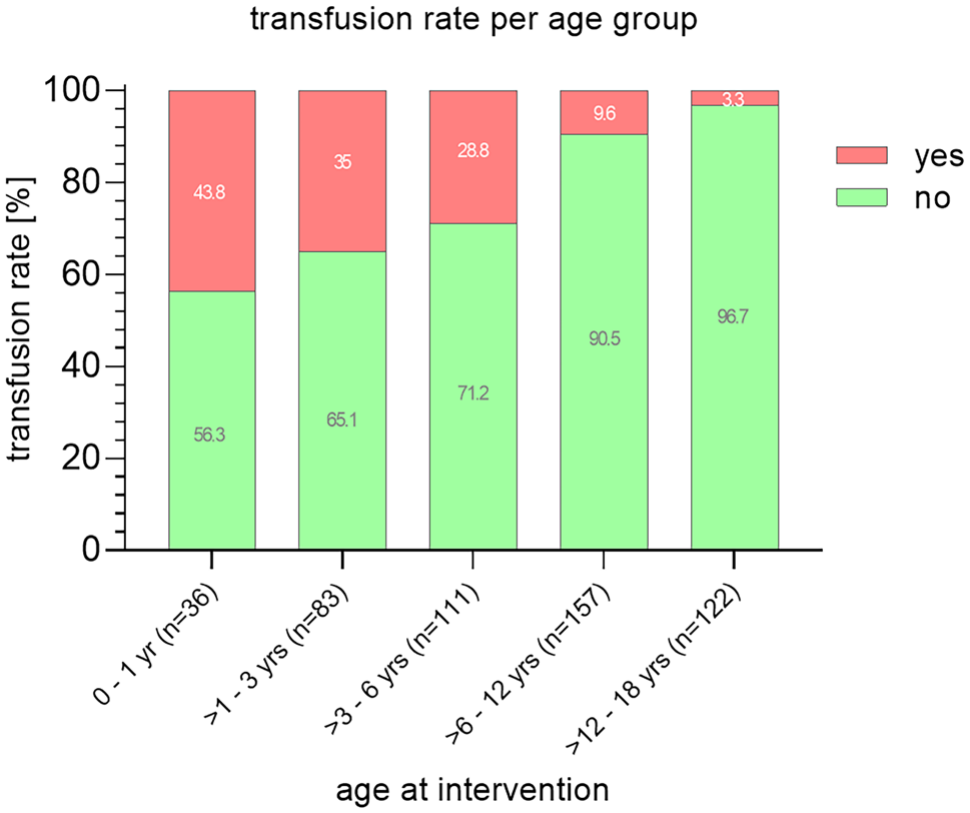
Age dependent rate for the need of transfusion showed an obvious correlation

Perioperative transfusions (123 erythrocyte concentrates, 111 fresh frozen plasma, and 7 platelet concentrates) were necessary in 94 of the 531 operations (17.7%). The transfusion rate decreased significantly with increasing age of patients (Fig. 3).

### Tumor localization and histology

**Fig. 4:**
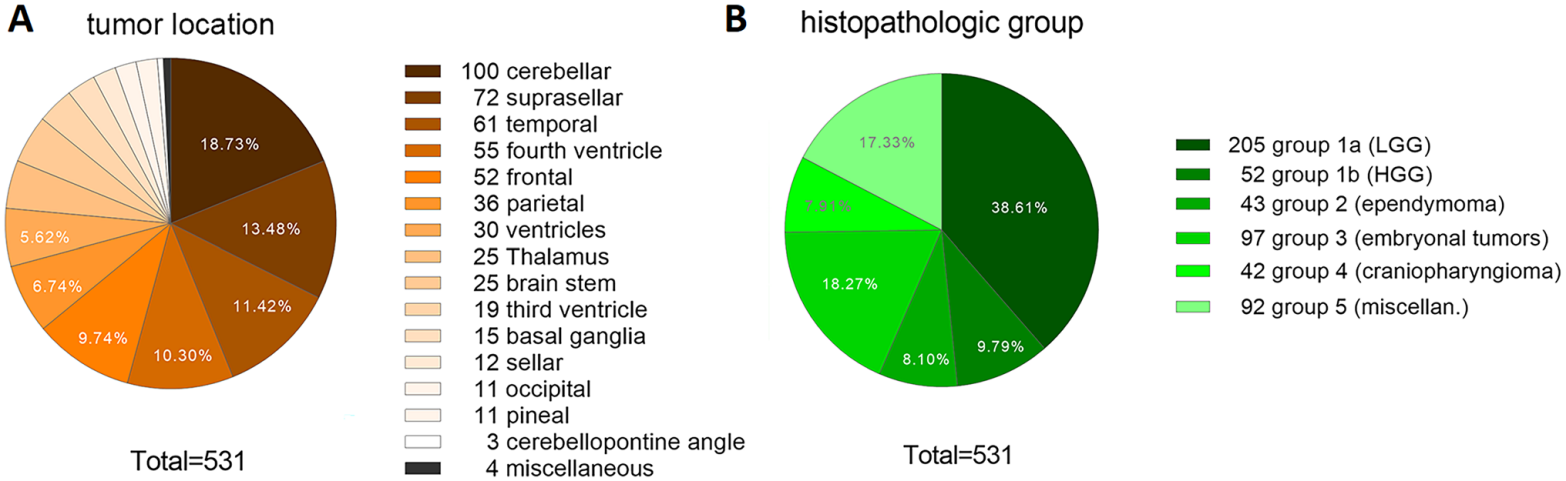
Distribution of tumor location (**A**) and group of histological diagnosis (**B**)

Main tumor approach at surgery was supratentorial in 346 surgical interventions (65.2%), while 185 surgeries addressed infratentorial tumors (34.8%) (Fig. 4A, Table 1, supplementary tables 1 and 2).

Two hundred five cases (38.6%) are filed into group 1a “LGG,” 52 cases (9.8%) into group 1b “HGG,” 43 cases (8.1%) into group 2 “ependymoma,” 97 cases (18.2%) into group 3 “embryonal tumors,” 42 cases (7.9%) into group 4 “craniopharyngioma,” and 92 cases (17.3%) into group 5 “other” (Fig. 4B, Table 1, more detailed histology subtypes are given in supplementary table 3).

According to the WHO classification 2016, 235 tumors displayed grade I (44.2%), 28 tumors grade II (5.3%), 71 tumors grade III (13.4%), and 129 tumors grade IV (24.3%). Sixty-eight histologic findings (12.8%) were not applicable to the WHO-grading scheme (e.g., Ewing sarcoma, rhabdomyosarcoma, hamartoma, tumor necrosis) [13]. Infants showed a higher percentage of malignant tumors (WHO I/II: 31% vs. WHO III/IV: 53%) in contrast to adolescents (WHO I/II: 58% vs. WHO III/IV: 28%, Fig. 4).

### Neurological outcome

**Fig. 5:**
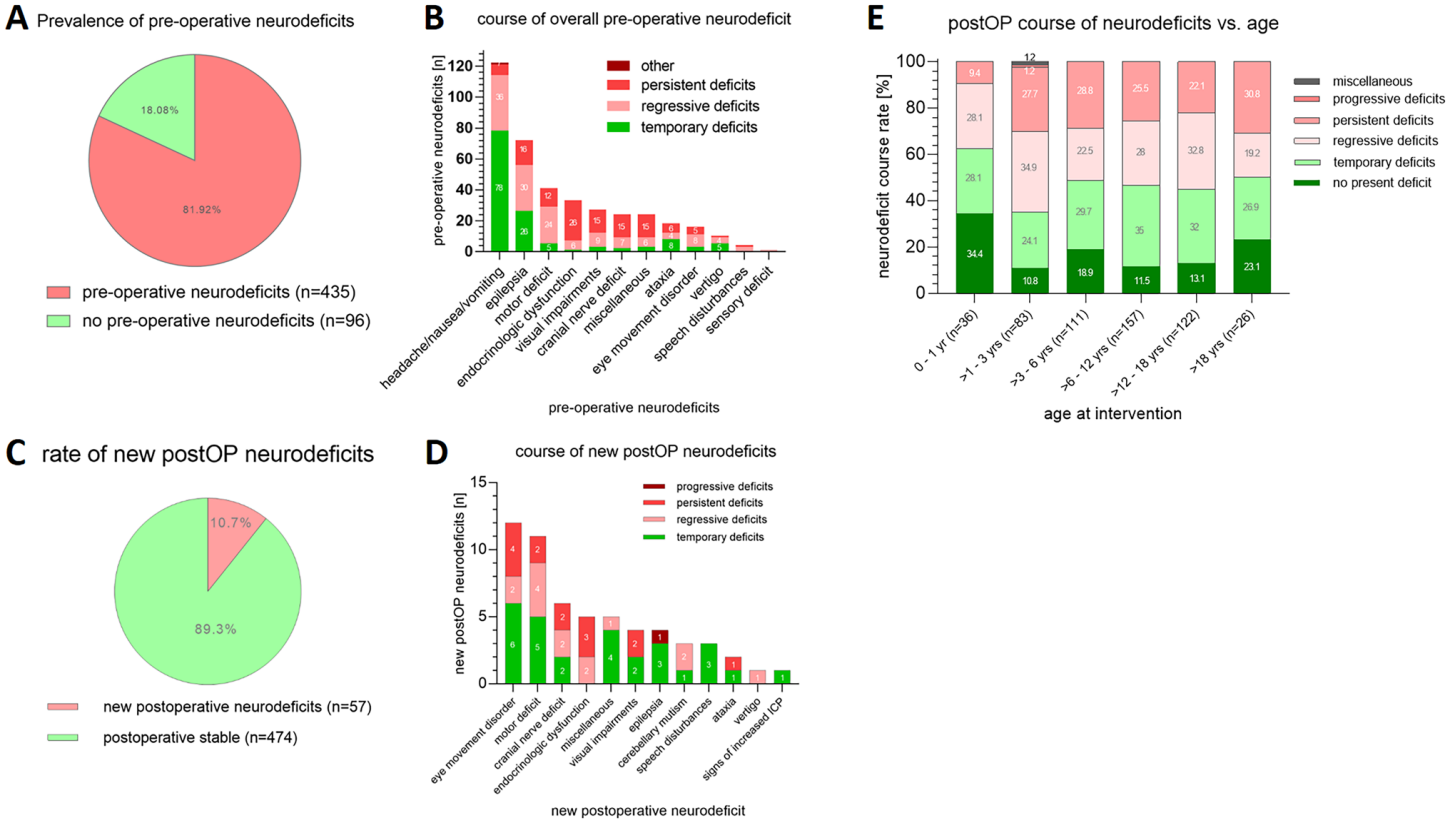
Preoperative neurological deficits were present in the vast majority of cases (**A**), which showed heterogeneous development over time after surgery (**B**). New neurological deficits after surgery were observed in 10.7% of cases (**C**), which developed postoperatively more favorable compared to preexisting neurological deficits with 5.3% resolving, 2.7% regressive, and 2.8% persistent deficits (**D**). No significant differences were seen in age dependent distribution of postoperative neurological deficits, however with relatively lower rates of progressive or persistent neurological deficits in infants at an age < 1 year and relatively higher rates in children with the age between 1 and 3 years (**E**)

Preoperative neurologic deficit was observed in 81.9% of the cases (*n* = 435; Table 2). Of these preexisting deficits, 34% disappeared after surgery (*n* = 148), while 35% (*n* = 152) ameliorated, and 30.3% persisted (*n* = 132) within 30 days after surgery (Table 2, Fig. 5A, B).

**Table 2:** Characterization of postoperative neurologic deficits divided in preexisting preoperative and newly developed impairments

**Neurologic deficits**	**Number of pre-op deficits (*n*)**	**Number of newly developed post-op deficits (*n*)**	**Total of deficits (*n*)**	**Rate of persist. deficits in deficits in total (%)**	**Percentage of persistent deficits (%)**	**Number of pre-op deficits in persist. deficits (*n*)**	**Rate of persist. deficits in pre-op deficits (%)**	**Rate of persist. deficits in newly devel. post-op deficits (%)**
**Endocrinologic dysfunction**	38	5	43	32/43 (74%)	74%	29/32	29/38 (76%)	3/5 (60%)
**Visual impairments**	29	4	33	19/33 (58%)	58%	17/19	17/29 (59%)	2/4 (50%)
**Cranial nerve deficit**	26	6	32	18/32 (56%)	56%	16/18	16/26 (61%)	2/6 (33%)
**Ataxia**	21	2	23	8/23 (35%)	35%	7/8	7/21 (33%)	1/2 (50%)
**Eye movement disorder**	16	12	28	9/28 (32%)	32%	5/9	5/16 (31%)	4/12 (33%)
**Motor deficit**	50	11	61	17/61 (28%)	28%	15/17	15/50 (30%)	2/11 (18%)
**Epilepsia**	79	4	83	16/83 (19%)	19%	16/16	16/79 (20%)	0/3 (0%)
**Speech disturbances**	4	3	7	1/7 (14%)	14%	1/1	1/4 (25%)	0/3 (0%)
**Vertigo**	10	1	11	1/11 (9%)	9%	1/1	1/10 (10%)	0/1 (0%)
**Headache/nausea/vomiting (signs of increased ICP)**	136	1	137	9/137 (7%)	7%	9/9	9/136 (6%)	0/1 (0%)
**Miscellaneous**	25	5	30	15/30 (50%)	50%	15/15	15/25 (60%)	0/5 (0%)
**Cereb. mutism**	0	3	3	0/3 (0%)	0%	0/0	n.a	0/3 (0%)
**Sensor deficit**	1	0	1	0/1 (0%)	0%	0/0	0/1 (0%)	n.a
**Total**	**435**	**57**	**492**	**145/492 (29%)**	**29%**	**131/145 (90%)**	**131/435 (30%)**	**14/57 (24%)**

Postoperatively, 474 cases (89.3%) displayed no new neurological deficit (Fig. 5C, D). The most frequent new postoperative deficit were eye movement disorders with 12 cases (2.3%), hemiparesis/motor deficits with 11 cases (2.0%), other cranial nerve deficits with 6 cases (1.1%), as well as endocrine dysfunctions with 5 cases (0.9%). No significant age-dependent differences of neurological deficits have been observed; however, infants seem to show less progressive or persistent neurological deficits.

The postoperative duration of the combined pre- and postoperative neurological deficits was temporary in 163 cases (30.7%), regressive in 152 cases (28.6%), and persistent in 133 cases (25.0%). One patient (0.2%) showed a new progressive postoperative neurological deficit (1.28 years old male with a biventricular tumor mass including cerebrospinal fluid (CSF) dissemination; the preexisting VIIth nerve palsy progressed after biopsy; diagnosis of atypical teratoid rhabdoid tumor (ATRT) was treated unsuccessfully by neo-adjuvant medical therapy. The patient deceased on 37th postoperative day.

In terms of permanent hydrocephalus management, 11.5% of cases (*n* = 61) had a CSF diversion (EVD/ETV/shunting system) prior to the tumor surgery, and 8.2% of cases (*n* = 44) were shunt dependent 90 days after surgery. Eleven cases had an endoscopic third ventriculostomy (ETV) done before surgery, and 7 cases received a new ETV within 90 days following tumor surgery. Fourteen cases received a new shunt system while it could be removed in 6 cases during 90 days after surgery. A more detailed overview is given in supplement Table 2. Differences in infratentorial versus supratentorial tumor location and hydrocephalus management are given in supplementary table 1.

### Surgical complications

A total of 35 cases (6.6%) showed perioperative complications and 14 of these needed subsequent surgeries. CSF collection or leakage was seen in 16 cases of which nine needed surgical revision, endoscopic intervention, or shunt placement. Seven cases could be handled conservatively. Postoperative hemorrhages were seen in 13 cases. Four of these required surgery; three patients needed hematoma evacuations, whereas one patient needed shunt treatment due to posthemorrhagic hydrocephalus. Nine cases were treated conservatively. Two cases of postoperative infections were successfully treated with antibiotic therapy. Other complications included a skin ulceration which needed surgical treatment, temporary hepatic dysfunction due to patient positioning, edematous pancreatitis, and persistent postoperative anemia, which were treated conservatively.

### Overall survival

Fifty-nine patients (20.1%) had inconsistent datasets for follow-up. Of those, 35 patients (11.9%) were further treated at other departments mainly due to the distance of referral (“superregional”: *n* = 4, “distant”: *n* = 31). Contact was lost to twenty-four patients (8.2%) after mean follow up of 1.6 years due to various reasons (group 1a “LGG”: 14 patients, group 1b “HGG”: 3 patients, group 2 “ependymoma”: 2 patients, group 3 “embryonal tumors”: 1 patient, group 4 “craniopharyngeoma”: 1 patient and group 5 “other”:3 patients; CR: *n* = 8; SD: *n* = 12; PD: *n* = 4).

In the remaining cohort of 200 patients, the follow-up time was 5.7 ± 2.9 years (range: 2.1–19.3 years, median 5.2 years, IQR 3.9 years). The survival in the entire cohort was 90.4%. Thirty-four patients (11.6%) died during the study period (Table 1). Time to death was 1.4 ± 1.3 years on average after surgery (range: 0–6.1 years, median 1.1 years, IQR 1.7 years) with minor variation in different tumor entities (group 1a “LGG”: 1.8 ± 1.2 years (*n* = 3), group 1b “HGG”: 1.4 ± 0.9 years (*n* = 7), group 2 “ependymoma”: 3.2 ± 2.9 years (*n* = 2), group 3 “embryonal tumors” 1.3 ± 1.2 years (*n* = 16), and group 5 “other” 1.1 ± 0.9 years (*n* = 6)).

The 30-day overall mortality rate was 1% (3 patients out of 293 patients). One patient died on the day of surgery (2.99 years old boy, postictal comatose state with dilated pupils on hospital admission. Cranial CT showed bifrontal CNS-PNET tumor mass and consecutive hydrocephalus. Subsequent emergency operation (N0) with tumor debulking during ongoing resuscitation was immediately initiated. Patient finally deceased during early postoperative phase). One patient died on the 26th postoperative day (3.1 years old boy with metastasized ATRT and right temporal mass resistant to chemo and radiation therapy), and one patient died on the 29th postoperative day (1.6 years old boy, metastasized medulloblastoma, resistant to medical tumor therapy).

### Radiological outcome

The mean time from last surgery to last available MRI was 2.9 ± 2.7 years (range: 0.02–9.5 years, median 2.2 years, IQR 4.2 years). Nevertheless, the MRI follow-up time of the last patient included in the study was 30 months. On these last available MRIs, 37.2% of the patients showed a complete remission, 36.5% displayed a stable disease, and 25.9% progressed.

## Discussion

We report on our neurosurgical experience taken from 20 years in patients with at least one brain tumor surgery between 01/2010 and 06/2017 at our dedicated pediatric neuro-oncologic center. The data reflects characteristics, challenges, and quality of care of an interdisciplinary approach to treat the heterogeneous group of children suffering from divers entities of brain tumors.

A good comparability of our data set and their consistency can be demonstrated with previously published cohort series from the last decade in terms of epidemiology, resection rate, transfusion rate, tumor localization, neuropathology, as well as pre- and postoperative deficits. The meta-analysis of those reflects an age range of 8.2–9.0 years [5–7, 19, 20] being comparable to our mean age of 8.3 years. The slight male predominance of patients (54.9% male) from our data is consistent with previously published data (range 50.4–60.0% male) [5, 6, 19–22]. Only a single publication showed an inversed ratio of 46.5% male and 53.5% female patients [7].

The primary tumor surgery in the patients’ history was performed in 84.9% in our department and is comparable to the studies of Neervort et al., Santos et al., und Lassen et al. (range 72.4–88.3%) [5–7]. The rate of 13.9% biopsies in our study is slightly lower than in other reports (16.7–16.9% biopsies) [5, 20].

In terms of preoperative neurological deficits (81.9% of cases), comparable publications showed a rather uniform distribution of clinical signs at presentation with increased ICP/headaches/vomiting being most frequent (47–51%) [20, 22] followed by seizures in 15.1–24% [20, 22]. Other symptoms include neurocognitive deficits 12.1–21% [20, 22] and visual deficits of 37% [22]. The clinically detectable neurological deficits are obviously tumor location- and age-dependent due to the patients’ neurologic development and thus communication/interaction skills. In the age group of infants, signs of an increased ICP (37.5%) are noted predominantly compared to motor deficits (up to 12%), cranial nerve deficits (8.2%), or endocrine dysfunctions (19.2%) as in older age groups.

Interestingly, we observed a difference in the distribution of preoperative deficits for primary operations in patients residing less than 50 km away from the hospital, with the type of deficits seen in patients living further away. Patients with acute symptoms such as signs of increased ICP or seizure disorders are more likely to be treated closer to their place of residence (e.g., occurrence of acute seizure disorders with 19.4% ≤ 50 km vs. 12.1% > 50 km). On the contrary, oligosymptomatic or chronically affected patients are able to travel a longer distance for treatment if that was the families’ aim (e.g., visual field deficits with 1.3% ≤ 50 km vs. 12.1% > 50 km).

In terms of tumor location at surgery, 65.2% cases received supratentorial and 34.8% infratentorial tumor approach in our study, while dissemination was seen in 16.1% of the cases, which is comparable with previous studies (supratentorial: 60–71.1%) [6, 7]. Others showed, however, a more balanced supra-/infratentorial distribution (supratentorial: 46.5–51%) [20, 22]. Differences in case interventions might be explained by the differing indications for surgery in the comparable series possibly being related to center or national surgical treatment policy.

Despite the various changes in the WHO CNS-tumor classification over time, the proportion of tumor entities remains constant. The three main tumor entities are represented similarly: glial tumors with 38.8–41%, embryonal tumors with 20–26.2%, and ependymal tumors with 7–8.9%. [5, 23]. This underlines the representative character of our study cohort within the population of German pediatric brain tumor patients.

According to measures for the extent of resection, reported rates vary among published studies depending on different classifications used. In our study, the tumor resection was rated by the combined neurosurgical and neuroradiological assessment [9]. A total (type I) or near total (type II) removal of the tumor was performed in 54.8% of initial surgeries, a partial removal (type III) in 31.2%, whereas 13.9% were biopsies (type IV). Differences for supratentorial and infratentorial tumors are shown in supplementary table 2. Modern treatment strategies including defensive resection technique to preserve quality of life as well as novel adjuvant therapy protocols have led to a substantial change towards less radical surgical strategies, especially in complex anatomical conditions. In addition, increasingly sensitive MRI imaging has changed the evaluation of the extent of resection [24]. In the German LGG registry, the complete/subtotal resection rate decreased from 54.4 to 32% between 1996 and 2018 [25]. Experience in pediatric neurosurgical brain tumor treatment and the role of surgery within an interdisciplinary approach have become the core of decision making for safe resection surgeries [26]. In this context, the “preoperative-intended” extent of resection is defined during the multidisciplinary team (MDT) conference and was compared with the “postoperative-achieved” extent of resection in this study. We observed a high conformity in the planned versus achieved extent of tumor resection (total (S1): 92.4%; rim (S2): 75.3%; partial (S3): 96.9%; biopsy (S4): 97.3%).

The current literature stresses the importance of complete tumor removal for the outcome in selected malignant tumor types, such as ependymoma [29], ATRT [28], and somehow also medulloblastoma [27]. This may be less relevant for benign tumors since non-space-occupying tumor remnants might not necessarily influence the overall survival of the patients. Even more, a complete removal might result in decreased neurological function, leading to an impaired quality of life. Especially in medulloblastoma cases, the rate of cerebellar mutism needs to be balanced against less aggressive surgeries as consequent adjuvant treatment with second look surgeries might be an alternative strategy.

This also applies to infants presenting with extensive malignant tumors as they pose a challenge for the operative team in order to manage the surgery safely. This is also reflected by the transfusion rate which clearly correlates with patients’ age (41.2% at 0–1 year vs. 3.3% at 12–18 years). Staged surgical approaches with neo-adjuvant chemotherapy have become a safe alternative to initial complete resection inheriting an elevated transfusion necessity. A recent publications state also a correlation of blood transfusion with patients’ age but not tumor entity [30]. Close interdisciplinary interaction during the operative intervention (e.g., anesthesiology, neurosurgery, and intensive care medicine) is essential to lower the transfusion rate [31] and the need for intensive care [32].

In terms of postoperative function, no additional morbidity was seen in 89.3% of patients. 81 of the cases (15.2%) did not show any neurologic deficit at any time point, neither preoperatively nor postoperatively. If deficits were observed, complete remission or regression of pre- and postoperative symptoms were seen in 69.3% of patients, while in 25% of the patients, there was no change in the neurologic deficits within 30 days after surgery. However, comparison of preoperative neurologic deficits as well as new postoperative neurologic deficits regarding postoperative persistence prove to be different. The likelihood of persistent neurologic deficit after surgery is higher for preoperatively existing deficits than new postoperative neurologic deficits (Table 2). Thus, new postoperative neurologic deficits show a time-limited, more benign course than preoperatively existing neurologic deficits (Fig. 4A–D). Comparisons of these results with other publications are rather difficult due to different terms of reporting: e.g. mild to severe deficits in 22.0% [6], long-term neurologic morbidity in 19% [5], and moderate to severe deficits in 12.4% [7] are stated. No conclusive publications are available for the pediatric population regarding the rate of new postoperative neurologic deficits so far. For quality measures, a standardized and detailed reporting form would be advantageous for future studies.

In addition, predisposing factors for neurologic deficits or quality of life effects may be more intensively studied on a center specific basis, as it has been reported for individual tumor entities e.g., craniopharyngioma [33] or for specific lesion locations, e.g., posterior fossa tumors [34].

The overall 30-day-mortality rate was 1% in our study. One patient died on the day of surgery due to fatal neurologic condition already present on admission to the hospital (histology: CNS-PNET). Two patients died at later time points within the 30 days period related to an uncontrollable oncologic tumor progression (ATRT and medulloblastoma). Thus, postoperative mortality rate was 0.3%, similar as reported earlier, 0–0.8% [5–7]; however, none of the patients died directly as a cause of surgical intervention.

## Conclusion

In this study, we display a pediatric neurosurgery brain tumor patient’s care from the perspective of an interdisciplinary neuro-oncological center in Germany. The study contributes to define challenges of dealing with the heterogeneity of pediatric brain tumor cases and quality indicators for brain tumor surgery in children in a dedicated pediatric neurosurgery setting. From our perspective, a consequent and experience driven strategy with reliable, multidisciplinary decision making on subsequent therapy is warranted to enhance the treatment quality of children with heterogeneous tumor entities to aim for long-term quality of survival.

### Supplementary Information

Below is the link to the electronic supplementary material.

Supplementary file1 (DOCX 33 KB)

## References

[1] Bhakta N, Force LM, Allemani C, Atun R, Bray F, Coleman MP, Steliarova-Foucher E, Frazier AL, Robison LL, Rodriguez-Galindo C, Fitzmaurice C (2019). Childhood cancer burden: a review of global estimates. Lancet Oncol.

[2] Mehta V, Chapman A, McNeely PD, Walling S, Howes WJ (2002). Latency between symptom onset and diagnosis of pediatric brain tumors: an Eastern Canadian geographic study. Neurosurgery.

[3] Keene DL, Hsu E, Ventureyra E (1999). Brain tumors in childhood and adolescence. Pediatr Neurol.

[4] Kleihues P, Burger PC, Scheithauer BW (1993). The new WHO classification of brain tumours. Brain Pathol.

[5] Neervoort FW, Van Ouwerkerk WJ, Folkersma H, Kaspers GJ, Vandertop WP (2010). Surgical morbidity and mortality of pediatric brain tumors: a single center audit. Child’s nervous system: ChNS: official journal of the International Society for Pediatric Neurosurgery.

[6] Santos MM, Faria CC, Miguéns J (2016). Pediatric central nervous system tumors: review of a single Portuguese institution. Child’s nervous system: ChNS: official journal of the International Society for Pediatric Neurosurgery.

[7] Lassen B, Helseth E, Egge A, Due-Tønnessen BJ, Rønning P, Meling TR (2012). Surgical mortality and selected complications in 273 consecutive craniotomies for intracranial tumors in pediatric patients. Neurosurgery.

[8] Tallen G, Resch A, Calaminus G, Wiener A, Leiss U, Pletschko T, Friedrich C, Langer T, Grabow D, Driever PH, Kortmann RD, Timmermann B, Pietsch T, Warmuth-Metz M, Bison B, Thomale UW, Krauss J, Mynarek M, von Hoff K, Ottensmeier H, Fruhwald M, Kramm CM, Temming P, Muller HL, Witt O, Kordes U, Fleischhack G, Gnekow A, Rutkowski S, German Paediatric Brain Tumour C (2015). Strategies to improve the quality of survival for childhood brain tumour survivors. Eur J Paediatr Neurol.

[9] Gnekow AK (1995). Recommendations of the brain tumor subcommittee for the reporting of trials. SIOP Brain Tumor Subcommittee. International Society of Pediatric Oncology. Med Pediatr Oncol.

[10] Gnekow AK, Kandels D, Tilburg CV, Azizi AA, Opocher E, Stokland T, Driever PH, Schouten-van Meeteren AYN, Thomale UW, Schuhmann MU, Czech T, Goodden JR, Warmuth-Metz M, Bison B, Avula S, Kortmann RD, Timmermann B, Pietsch T, Witt O (2019). SIOP-E-BTG and GPOH guidelines for diagnosis and treatment of children and adolescents with low grade glioma. Klin Padiatr.

[11] Rutkowski S, Modena P, Williamson D, Kerl K, Nysom K, Pizer B, Bartels U, Puget S, Doz F, Michalski A, von Hoff K, Chevignard M, Avula S, Murray MJ, Schonberger S, Czech T, Schouten-van Meeteren AYN, Kordes U, Kramm CM, van Vuurden DG, Hulleman E, Janssens GO, Solanki GA, van Veelen MC, Thomale U, Schuhmann MU, Jones C, Giangaspero F, Figarella-Branger D, Pietsch T, Clifford SC, Pfister SM, Van Gool SW (2018). Biological material collection to advance translational research and treatment of children with CNS tumours: position paper from the SIOPE Brain Tumour Group. Lancet Oncol.

[12] Louis DN, Ohgaki H, Wiestler OD, Cavenee WK, Burger PC, Jouvet A, Scheithauer BW, Kleihues P (2007). The 2007 WHO classification of tumours of the central nervous system. Acta Neuropathol.

[13] Louis DN, Perry A, Reifenberger G, von Deimling A, Figarella-Branger D, Cavenee WK, Ohgaki H, Wiestler OD, Kleihues P, Ellison DW (2016). The 2016 World Health Organization Classification of Tumors of the Central Nervous System: a summary. Acta Neuropathol.

[14] Cotter JA, Viaene AN, Santi M, Hawkins C, Judkins AR (2022). A practical approach to the evaluation and diagnosis of pediatric CNS tumors. Pediatr Dev Pathol.

[15] Gritsch S, Batchelor TT, Gonzalez Castro LN (2022). Diagnostic, therapeutic, and prognostic implications of the 2021 World Health Organization classification of tumors of the central nervous system. Cancer.

[16] Komori T (2022) The 2021 WHO classification of tumors, 5th edition, central nervous system tumors: a short review. Brain Nerve 74: 803–809 10.11477/mf.1416202124 35676215

[17] Avula S, Peet A, Morana G, Morgan P, Warmuth-Metz M, Jaspan T, European Society for paediatric oncology -brain tumour imaging G (2021). European Society for Paediatric Oncology (SIOPE) MRI guidelines for imaging patients with central nervous system tumours. Child’s nervous system: ChNS: official journal of the International Society for Pediatric Neurosurgery.

[18] Bauer MATC, Kraus R, Rüggeberg J, Wardemann K, Müller P, Taube C, Diemer M, Schuster M (2020) Glossar perioperativer Prozesszeiten und Kennzahlen. Version 2020. Anästhesiologie & Intensivmedizin 61

[19] Moiyadi AV, Shetty P (2012). Feasibility of repeat surgery for pediatric brain tumors: an objective assessment of perioperative outcomes. J Neurosurg Pediatr.

[20] Bauchet L, Rigau V, Mathieu-Daude H, Fabbro-Peray P, Palenzuela G, Figarella-Branger D, Moritz J, Puget S, Bauchet F, Pallusseau L, Duffau H, Coubes P, Tretarre B, Labrousse F, Dhellemmes P, Societe Francaise de Neurochirurgie P, Societe Francaise de N, Association des Neuro-Oncologues d’'Expression F (2009). Clinical epidemiology for childhood primary central nervous system tumors. J Neurooncol.

[21] Ostrom QT, Gittleman H, Truitt G, Boscia A, Kruchko C, Barnholtz-Sloan JS (2018). CBTRUS statistical report: primary brain and other central nervous system tumors diagnosed in the United States in 2011–2015. Neuro Oncol.

[22] Stocco C, Pilotto C, Passone E, Nocerino A, Tosolini R, Pusiol A, Cogo P (2017). Presentation and symptom interval in children with central nervous system tumors. A single-center experience. Child’s nervous system: ChNS: official journal of the International Society for Pediatric Neurosurgery.

[23] Kaatsch P, Rickert CH, Kuhl J, Schuz J, Michaelis J (2001). Population-based epidemiologic data on brain tumors in German children. Cancer.

[24] Steinbok P, Mangat JS, Kerr JM, Sargent M, Suryaningtyas W, Singhal A, Cochrane D (2013). Neurological morbidity of surgical resection of pediatric cerebellar astrocytomas. Child's nervous system: ChNS: official journal of the International Society for Pediatric Neurosurgery.

[25] Gnekow AK, Kandels D, Pietsch T, Bison B, Warmuth-Metz M, Thomale UW, Kortmann RD, Timmermann B, Driever PH, Witt O, Schmidt R, Spix C (2021). Doubling recruitment of pediatric low-grade glioma within two decades does not change outcome - report from the German LGG studies. Klin Padiatr.

[26] Maharaj A, Manoranjan B, Verhey LH, Fleming AJ, Farrokhyar F, Almenawer S, Singh SK, Yarascavitch B (2019). Predictive measures and outcomes of extent of resection in juvenile pilocytic astrocytoma. J Clin Neurosci.

[27] Thompson EM, Bramall A, Herndon JE, Taylor MD, Ramaswamy V (2018). The clinical importance of medulloblastoma extent of resection: a systematic review. J Neurooncol.

[28] Richards A, Ved R, Murphy C, Hennigan D, Kilday JP, Kamaly-Asl I, Mallucci C, Bhatti I, Patel C, Leach P (2020). Outcomes with respect to extent of surgical resection for pediatric atypical teratoid rhabdoid tumors. Child’s nervous system: ChNS: official journal of the International Society for Pediatric Neurosurgery.

[29] Snider CA, Yang K, Mack SC, Suh JH, Chao ST, Merchant TE, Murphy ES (2018) Impact of radiation therapy and extent of resection for ependymoma in young children: a population-based study. Pediatr Blood Cancer 65 10.1002/pbc.26880 29115718

[30] Vassal O, Desgranges FP, Tosetti S, Burgal S, Dailler F, Javouhey E, Mottolese C, Chassard D (2016). Risk factors for intraoperative allogeneic blood transfusion during craniotomy for brain tumor removal in children. Paediatr Anaesth.

[31] Goobie SM, Haas T (2014). Bleeding management for pediatric craniotomies and craniofacial surgery. Paediatr Anaesth.

[32] Piastra M, Di Rocco C, Caresta E, Zorzi G, De Luca D, Caldarelli M, La Torre G, Conti G, Antonelli M, Eaton S, Pietrini D (2008). Blood loss and short-term outcome of infants undergoing brain tumour removal. J Neurooncol.

[33] Drapeau A, Walz PC, Eide JG, Rugino AJ, Shaikhouni A, Mohyeldin A, Carrau RL, Prevedello DM (2019). Pediatric craniopharyngioma. Child’s nervous system: ChNS: official journal of the International Society for Pediatric Neurosurgery.

[34] Tamburrini G, Frassanito P, Chieffo D, Massimi L, Caldarelli M, Di Rocco C (2015). Cerebellar mutism. Child’s nervous system: ChNS: official journal of the International Society for Pediatric Neurosurgery.

