# Supplementary material for: Neurosurgical treatment of pediatric brain tumors - results from a single center multidisciplinary setup
Source: Childs Nerv Syst. 2023 Sep 21;40(2):381–93. doi: 10.1007/s00381-023-06123-8 (PMC10837233; doi:10.1007/s00381-023-06123-8)
Supplement: Supplementary file 1 — Supplementary file1 (DOCX 33 KB) [file 381_2023_6123_MOESM1_ESM.docx]

**supplement table 1:** detailed data overview for tumor subgroups group 1a-5

|  |  |  |  |  |  |  |  |
| --- | --- | --- | --- | --- | --- | --- | --- |
| **item** | | **group 1a** | **group 1b** | **group 2** | **group 3** | **group 4** | **group 5** |
| **follow-up time span** | | 3.46a  (± 2.73) | 2.44a  (± 2.54) | 2.31a  (± 2.29) | 2.69a  (± 3.03) | 2.53a  (± 2.38) | 3.57a  (± 2.73) |
|  | |  |  |  |  |  |  |
|  | | n (%) | n (%) | n (%) | n (%) | n (%) | n (%) |
| **total** | | 205 | 52 | 43 | 97 | 42 | 92 |
| **tumor location** | | | | | | | |
| supratentorial | | 129 (62.9%) | 45 (86.5%) | 16 (37.2%) | 41 (42.3%) | 42 (100%) | 73 (79.3%) |
| infratentorial | | 76 (37.1%) | 7 (13.5%) | 27 (62.8%) | 56 (57.7%) | 0 (0%) | 19 (20.7%) |
| **extent of resection classification** | |  |  |  |  |  |  |
| …..type I (S1/R1) | | 59 (28.8%) | 17 (32.7%) | 21 (48.8%) | 26 (26.8%) | 3 (7.1%) | 32 (34.8%) |
| …..type IIa (S2/R1) | | 0 (0%) | 0 (0%) | 1 (2.3%) | 5 (5.2%) | 0 (0%) | 0 (0%) |
| …..type IIb (S1/R2) | | 27 (13.2%) | 10 (19.2%) | 6 (14.0%) | 8 (8.2%) | 2 (4.8%) | 7 (7.6%) |
| …..type IIc (S2/R2) | | 19 (9.3%) | 5 (9.6%) | 9 (20.9%) | 14 (14.4%) | 15 (35.7%) | 5 (5.4%) |
| …..type III (S123/R3) | | 69 (33.7%) | 16 (30.8%) | 6 (14.0%) | 29 (29.9%) | 20 (47.6%) | 26 (28.3%) |
| …..biopsy (S4/R4) | | 31 (15.1%) | 4 (7.7%) | 0 (0.0%) | 15 (15.5%) | 2 (4.8%) | 22 (23.9%) |
| **neurologic status** | | | | | | | |
| preop. neuro deficit | | 180 (87.8%) | 38 (73.1%) | 31 (72.1%) | 78 (80.4%) | 42 (100.0%) | 66 (71.7%) |
| new postop. neuro deficit | | 22 (10.7%) | 8 (15.4%) | 4 (9.3%) | 11 (11.3%) | 3 (7.1%) | 9 (9.8%) |
| **course of neurologic deficits** | | | | | | | |
| no neuro deficit | | 21 (10.2%) | 12 (23.1%) | 9 (20.9%) | 16 (16.5%) | 0 (0%) | 23 (25.0%) |
| temporary deficit | | 75 (36.6%) | 13 (25.0%) | 13 (30.2%) | 32 (33.0%) | 5 (11.9%) | 25 (27.2%) |
| regressive deficit | | 64 (31.2%) | 13 (25.0%) | 8 (18.6%) | 29 (29.9%) | 14 (33.3%) | 25 (27.2%) |
| persistent | | 45 (22.0%) | 14 (26.9%) | 13 (30.2%) | 19 (19.6%) | 23 (54.8%) | 19 (20.7%) |
| progressive deficit | | 0 (0%) | 0 (0%) | 0 (0%) | 1 (1.1%) | 0 (0%) | 0 (0%) |
| **follow-up status** | | | | | | | |
| complete remission (CR) | | 46 (35.9%) | 8 (29.6%) | 9 (42.9%) | 13 (24.5%) | 3 (21.4%) | 21 (42.0%) |
| stable disease (SD) | | 61 (47.7%) | 8 (29.6%) | 9 (42.9%) | 17 (32.1%) | 7 (50.0%) | 21 (42.0%) |
| progressive disease (PD) | | 18 (14.1%) | 3 (11.1%) | 1 (4.8%) | 8 (15.1%) | 4 (28.6%) | 2 (4.0%) |
| death | | 3 (2.3%) | 8 (29.6%) | 2 (9.5%) | 15 (28.3%) | 0 (0%) | 6 (12.0%) |

**supplement table 2:** detailed overview for supra- versus infratentorial tumor location

| **item** | **supratentorial** | **infratentorial** |
| --- | --- | --- |
| **follow-up time span** (from last surgery to end of study) | 2.93a (±2.56) | 3.43a (±2.97) |
|  |  |  |
|  | n (%) | n (%) |
| **total** | 346 (65.2%) | 185 (34.8%) |
| **tumor group** | | |
| group 1a (LGG) | 129 (37.3%) | 76 (41.1%) |
| group 1b (HGG) | 45 (13.0%) | 7 (3.8%) |
| group 2 (ependymoma) | 16 (4.6%) | 27 (14.6%) |
| group 3 (embryonal) | 41 (11.8%) | 56 (30.3%) |
| group 4 (craniopharyng.) | 42 (12.1%) | 0 (0%) |
| group 5 (others) | 73 (21.1%) | 19 (10.3%) |
| **extent of resection classification** | | |
| type I (S1/R1) | 95 (27.5%) | 63 (34.1%) |
| type IIa (S2/R1) | 0 (0%) | 6 (3.2%) |
| type IIb (S1/R2) | 35 (10.1%) | 25 (13.5%) |
| type IIc (S2/R2) | 46 (13.3%) | 21 (11.4%) |
| type III (S123/R3) | 114 (32.9%) | 52 (28.1%) |
| biopsy (S4/R4) | 56 (16.2%) | 18 (9.7%) |
| **neurologic status** | | |
| no preop neuro deficit | 60 (17.3%) | 36 (19.5%) |
| preop. neuro deficit | 286 (82.7%) | 149 (80.5%) |
| new postop. neuro deficit | 32 (9.2%) | 26 (14.1%) |
| **course of neurologic deficits** | | |
| no neuro deficit | 53 (15.4%) | 28 (15.1%) |
| temporary deficit | 93 (27.0%) | 70 (37.8%) |
| regressive deficit | 98 (28.4%) | 54 (29.2%) |
| persistent | 100 (29.0%) | 33 (17.8%) |
| progressive deficit | 1 (0.3%) | 0 (0%) |
| **HC management** | | |
| pre-existing EVDs | 4 (1.2%) | 5 (2.7%) |
| pre-existing ETVs | 11 (3.2%) | 10 (5.4%) |
| pre-existing shunts | 27 (7.8%) | 4 (2.2%) |
| explanted systems | 6 (1.7%) | 3 (1.6%) |
| newly ETVs | 7 (2.0%) | 6 (3.2%) |
| newly implanted shunts | 14 (4.0%) | 8 (4.3%) |
| ETVs in place total | 18 (5.2%) | 16 (8.6%) |
| shunts in place total | 35 (10.1%) | 9 (4.9%) |
| CSF diversion dependency | 53 (15.3%) | 25 (13.5%) |
| **follow-up status** | | |
| complete remission (CR) | 58 (32.6%) | 42 (36.5%) |
| stable disease (SD) | 79 (44.4%) | 44 (38.3%) |
| progressive disease (PD) | 22 (12.4%) | 14 (12.2%) |
| death | 19 (10.7%) | 15 (13.0%) |

**supplement table 3:** detailed neuropathological tumor subtypes

| **item** | **n (%)** |
| --- | --- |
| **group 1a (LGG)** | **205** |
| pilocytic astrocytoma | 135 (65.9%) |
| ganglioglioma | 30 (14.6%) |
| dysembryoplastic neuroepithelial tumor (DNT) | 9 (4.4%) |
| pilomyxoid astrocytoma | 6 (2.9%) |
| astrocytoma | 6 (2.9%) |
| rosette-forming glioneuronal tumor (RGNT) | 3 (1.5%) |
| others | 16 (7.8%) |
| **group 1b (HGG)** | **52** |
| glioblastoma multiforme | 19 (36.5%) |
| anaplastic astrocytoma | 14 (26.9%) |
| anaplastic oligoastrocytoma | 7 (13.5%) |
| anaplastic pleomorphic xanthoastrocytoma | 4 (7.7%) |
| others | 8 (15.4%) |
| **group 2 (ependymoma)** | **43** |
| ependymoma | 43 (100%) |
| **group 3 (embryonal)** | **97** |
| medulloblastoma | 60 (61.9%) |
| atypical teratoid/rhabdoid tumor (ATRT) | 32 (33.0%) |
| others | 5 (5.2%) |
| **group 4 (craniopharyngeoma)** | **42** |
| craniopharyngeoma | 42 (100%) |
| **group 5 (others)** | **92** |
| plexus tumor | 15 (16.3%) |
| sarcoma | 13 (14.1%) |
| pineal region tumor | 9 (9.8%) |
| hamartoma | 6 (6.5%) |
| neurofibroma/neurinoma | 5 (5.4%) |
| CNS PNET | 8 (8.7%) |
| malignant melanoma | 2 (2.2%) |
| mature teratoma | 2 (2.2%) |
| others | 32 (34.8%) |
| **total** | **531** |
